# Supplementary material for: PUFA stabilizes a conductive state of the selectivity filter in IKs channels
Source: eLife. 2024 Oct 31;13:RP95852. doi: 10.7554/eLife.95852 (PMC11527429; doi:10.7554/eLife.95852)
Supplement: Figure 1—source data 1. [file elife-95852-fig1-data1.docx]

| **WT** | **Control** | **0.2 µM** | **0.7 µM** | **2 µM** | **7 µM** | **20 µM** |
| --- | --- | --- | --- | --- | --- | --- |
| Gmax/Gmax0 | 1 | 1.40±0.11 | 1.62±0.15 | 1.87±0.16 | 2.25±0.20 | 2.40±0.28 |
| ∆V0.5 | 0 | 0.70± 1 | -1.5±1 | -7.7±2 | -24±2.3 | -24±6 |
