# Supplementary material for: PUFA stabilizes a conductive state of the selectivity filter in IKs channels
Source: eLife. 2024 Oct 31;13:RP95852. doi: 10.7554/eLife.95852 (PMC11527429; doi:10.7554/eLife.95852)
Supplement: Figure 3—source data 1. [file elife-95852-fig3-data1.docx]

| **Gmax/Gmax0** | **Control** | **0.2 µM** | **0.7 µM** | **2 µM** | **7 µM** | **20 µM** |
| --- | --- | --- | --- | --- | --- | --- |
| D301E | 1 | 0.7±0.13 | 0.7±0.14 | 0.7±0.17 | 0.90±0.20 | 0.72±0.12 |
| WT | 1 | 1.40±0.11 | 1.62±0.15 | 1.87±0.16 | 2.25±0.20 | 2.40±0.28 |
|  |  |  |  |  |  |  |
