# Supplementary material for: PUFA stabilizes a conductive state of the selectivity filter in IKs channels
Source: eLife. 2024 Oct 31;13:RP95852. doi: 10.7554/eLife.95852 (PMC11527429; doi:10.7554/eLife.95852)
Supplement: Figure 5—source data 1. [file elife-95852-fig5-data1.docx]

|  |  |  |  |  |  |  |
| --- | --- | --- | --- | --- | --- | --- |
| **Gmax/Gmax0** | **Control** | **0.2 µM** | **0.7 µM** | **2 µM** | **7 µM** | **20 µM** |
| **WT** | 1 | 1.40±0.11 | 1.62±0.15 | 1.87±0.16 | 2.25±0.20 | 2.40±0.28 |
| T309S | 1 | 1.3±0.06 | 1.24±0.03 | 1.38±0.08 | 1.63±0.15 | 1.63±0.02 |
| Y315F | 1 | 0.9±0.11 | 0.7±0.12 | 0.6±0.14 | 0.7±0.2 | 0.7±0.2 |
| D317E | 1 | 1.06±0.05 | 1.2±0.11 | 1.4±0.18 | 1.5±0.21 | 1.5±0.25 |
| T312C | 1 | 1.12±0.07 | 1.4±0.33 | 1.5±0.33 | 2.1±0.67 | 2.49±0.98 |
| I313S | 1 | 1.5±0.13 | 1.5±0.14 | 1.6±0.12 | 1.9±0.12 | 2.18±0.05 |
| P320L | 1 | 1.03±0.08 | 1.03±0.10 | 1.02±0.12 | 1.15±0.15 | 1.25±0.21 |
