# Supplementary material for: PUFA stabilizes a conductive state of the selectivity filter in IKs channels
Source: eLife. 2024 Oct 31;13:RP95852. doi: 10.7554/eLife.95852 (PMC11527429; doi:10.7554/eLife.95852)
Supplement: Figure 5—figure supplement 1—source data 1. [file elife-95852-fig5-figsupp1-data1.docx]

| **∆V0.5 (mV)** | **Control** | **0.2 µM** | **0.7 µM** | **2 µM** | **7 µM** | **20 µM** |
| --- | --- | --- | --- | --- | --- | --- |
| Y315F | 0 | -1.8±1 | -2.4±1.5 | -5.2±1.5 | -16.4±2.4 | -21.2±5 |
| WT | 0 | 0.70± 1 | -1.5±1 | -7.7±2 | -24±2.3 | -24±6 |
