# Supplementary material for: PUFA stabilizes a conductive state of the selectivity filter in IKs channels
Source: eLife. 2024 Oct 31;13:RP95852. doi: 10.7554/eLife.95852 (PMC11527429; doi:10.7554/eLife.95852)
Supplement: Figure 5—figure supplement 2—source data 1. [file elife-95852-fig5-figsupp2-data1.docx]

| GV values for D301E |
| --- |

| Voltage step (mV) | Control | SEM | 20 µM | SEM |
| --- | --- | --- | --- | --- |
| -100 | -0.22 | 0.09 | -0.13 | 0.06 |
| -80 | -0.21 | 0.09 | -0.14 | 0.06 |
| -60 | -0.20 | 0.08 | -0.12 | 0.06 |
| -40 | -0.20 | 0.07 | -0.06 | 0.59 |
| -20 | -0.12 | 0.05 | 0.11 | 0.038 |
| 0 | 0.09 | 0.03 | 0.43 | 0.041 |
| 20 | 0.42 | 0.02 | 0.70 | 0.079 |
| 40 | 0.7 | 0.02 | 0.87 | 0.116 |
| 60 | 0.9 | 0.02 | 0.97 | 0.113 |
| 80 | 1 | 0 | 1 | 0 |

| GV values for T309S |
| --- |

| Voltage step (mV) | Control | SEM | 20 | SEM |
| --- | --- | --- | --- | --- |
| -100 | -0.33 | 0.11 | -0.50 | 0.09 |
| -80 | -0.33 | 0.11 | -0.52 | 0.11 |
| -60 | -0.32 | 0.10 | -0.49 | 0.11 |
| -40 | -0.30 | 0.11 | -0.29 | 0.07 |
| -20 | -0.25 | 0.10 | 0.27 | 0.13 |
| 0 | -0.04 | 0.09 | 0.93 | 0.16 |
| 20 | 0.30 | 0.08 | 1.50 | 0.16 |
| 40 | 0.66 | 0.05 | 1.85 | 0.13 |
| 60 | 0.91 | 0.02 | 1.93 | 0.08 |
| 80 | 1.00 | 0 | 1.70 | 0.13 |

| GV values for Y315F |
| --- |

| Voltage (mV) | Control | SEM | 20 µM | SEM |
| --- | --- | --- | --- | --- |
| -100 | -0.54 | 0.08 | -0.30 | 0.04 |
| -80 | -0.56 | 0.09 | -0.29 | 0.03 |
| -60 | -0.53 | 0.08 | -0.27 | 0.04 |
| -40 | -0.49 | 0.08 | -0.22 | 0.04 |
| -20 | -0.40 | 0.09 | -0.07 | 0.09 |
| 0 | -0.13 | 0.06 | 0.24 | 0.11 |
| 20 | 0.26 | 0.03 | 0.54 | 0.10 |
| 40 | 0.60 | 0.04 | 0.76 | 0.12 |
| 60 | 0.88 | 0.04 | 0.86 | 0.13 |
| 80 | 1.00 | 0 | 0.86 | 0.13 |

| GV values for D317E |
| --- |

| Voltage (mV) | Control | SEM | 20 µM | SEM |
| --- | --- | --- | --- | --- |
| -100 | -0.25 | 0.08 | -0.09 | 0.08 |
| -80 | -0.25 | 0.08 | -0.08 | 0.08 |
| -60 | -0.23 | 0.08 | -0.02 | 0.77 |
| -40 | -0.23 | 0.08 | 0.21 | 0.10 |
| -20 | -0.15 | 0.77 | 0.76 | 0.15 |
| 0 | 0.04 | 0.07 | 1.28 | 0.20 |
| 20 | 0.36 | 0.05 | 1.63 | 0.25 |
| 40 | 0.66 | 0.02 | 1.84 | 0.29 |
| 60 | 0.89 | 0.00 | 1.91 | 0.31 |
| 80 | 1.00 | 0 | 1.87 | 0.31 |

| GV values for T312C |
| --- |

| Voltage (mV) | Control | SEM | 20 µM | SEM |
| --- | --- | --- | --- | --- |
| -100 | -0.03 | 0.06 | -0.04 | 0.05 |
| -80 | -0.02 | 0.07 | -0.04 | 0.04 |
| -60 | -0.01 | 0.06 | -0.03 | 0.03 |
| -40 | -0.02 | 0.06 | 0.01 | 0.02 |
| -20 | -0.003 | 0.07 | 0.23 | 0.11 |
| 0 | 0.07 | 0.06 | 0.70 | 0.33 |
| 20 | 0.25 | 0.06 | 1.27 | 0.60 |
| 40 | 0.52 | 0.05 | 1.76 | 0.82 |
| 60 | 0.82 | 0.02 | 2.04 | 0.92 |
| 80 | 1.00 | 0 | 1.98 | 0.89 |

| GV values for I313S |
| --- |

| Voltage (mV) | Control | SEM | 20 µM | SEM |
| --- | --- | --- | --- | --- |
| -100 | -0.11 | 0.14 | -0.09 | 0.17 |
| -80 | -0.12 | 0.15 | -0.08 | 0.17 |
| -60 | -0.12 | 0.15 | 0,003 | 0.13 |
| -40 | -0.10 | 0.13 | 0.40 | 0.13 |
| -20 | -0.01 | 0.09 | 0.94 | 0.33 |
| 0 | 0.15 | 0.09 | 1.45 | 0.38 |
| 20 | 0.38 | 0.12 | 1.99 | 0.30 |
| 40 | 0.61 | 0.12 | 2.4 | 0.23 |
| 60 | 0.84 | 0.06 | 2.5 | 0.25 |
| 80 | 1.00 | 0 | 2.5 | 0.23 |

| GV values for P320L |
| --- |

| Voltage (mV) | Control | SEM | 20 µM | SEM |
| --- | --- | --- | --- | --- |
| -100 | -0.11 | 0.11 | -0.03 | 0.06 |
| -80 | -0.10 | 0.11 | -0.04 | 0.06 |
| -60 | -0.09 | 0.10 | -0.01 | 0.06 |
| -40 | -0.07 | 0.10 | 0.09 | 0.08 |
| -20 | -0.01 | 0.09 | 0.36 | 0.14 |
| 0 | 0.16 | 0.06 | 0.71 | 0.18 |
| 20 | 0.45 | 0.03 | 1.01 | 0.20 |
| 40 | 0.72 | 0.01 | 1.23 | 0.21 |
| 60 | 0.91 | 0.007 | 1.30 | 0.19 |
| 80 | 1.00 | 0 | 1.30 | 0.17 |
