# Supplementary material for: PUFA stabilizes a conductive state of the selectivity filter in IKs channels
Source: eLife. 2024 Oct 31;13:RP95852. doi: 10.7554/eLife.95852 (PMC11527429; doi:10.7554/eLife.95852)
Supplement: Figure 6—source data 1. [file elife-95852-fig6-data1.docx]

| **% Non-Empty Sweeps** | **Control** | **20 µM** |
| --- | --- | --- |
| Y315F | 10.9 | 8.3 |
| WT | 20.5 | 49.5 |
